# A sublingual nanofiber vaccine to prevent urinary tract infections

**DOI:** 10.1126/sciadv.abq4120

**Published:** 2022-11-23

**Authors:** Sean H. Kelly, Nicole L. Votaw, Benjamin J. Cossette, Yaoying Wu, Shamitha Shetty, Lucas S. Shores, Luqman A. Issah, Joel H. Collier

**Affiliations:** Department of Biomedical Engineering, Duke University, Durham, NC, USA.

## Abstract

Urinary tract infections (UTIs) are a major public health problem affecting millions of individuals each year. Recurrent UTIs are managed by long-term antibiotic use, making the alarming rise of antibiotic resistance a substantial threat to future UTI treatment. Extended antibiotic regimens may also have adverse effects on the microbiome. Here, we report the use of a supramolecular vaccine to provide long-term protection against uropathogenic *Escherichia coli*, which cause 80% of uncomplicated UTIs. We designed mucus-penetrating peptide-polymer nanofibers to enable sublingual (under the tongue) vaccine delivery and elicit antibody responses systemically and in the urogenital tract. In a mouse model of UTI, we demonstrate equivalent efficacy to high-dose oral antibiotics but with significantly less perturbation of the gut microbiome. We also formulate our vaccine as a rapid-dissolving sublingual tablet that raises response in mice and rabbits. Our approach represents a promising alternative to antibiotics for the treatment and prevention of UTIs.

## Teaser

A nanofiber vaccine for urinary tract infections perturbs the gut microbiome less than antibiotics.

## INTRODUCTION

More than half of all women experience a urinary tract infection (UTI) in their lifetime, and UTIs are a persistent complication of indwelling urinary catheters (*1*, *2*). Those suffering with recurrent UTIs [more than three infections in a 1-year period (*3*)] experience considerable loss in their quality of life and are burdened with increased health care costs (*4*, *5*). Recurrent UTIs are managed by long-term antibiotic prophylaxis, although this is not recommended until other behavioral or non-antibiotic options have been attempted (*6*, *7*). In addition to drug-specific adverse effects (*8*), the prolonged use of antibiotics alters the patient’s microbiota (*9*). Antibiotic treatment alters microbes’ metabolic activity, gene expression, and protein synthesis, in addition to reducing the diversity of the microbiota as a whole and favoring resistant populations (*10*).

The influence of antibiotics on the microbiome is reflected in the alarming rise of antibacterial resistance (*11*). Modeling has forecast that, by 2050, current practices would result in 10 million additional deaths per year by infection and a global economic cost of $100 trillion (*12*, *13*). Antibiotic resistance compounds the effects already associated with prolonged antibiotic use, making it likely that safe, effective treatment and prevention of UTIs will become increasingly challenging. Uropathogenic *Escherichia coli* (UPEC), which cause about 80% of uncomplicated UTIs, have become increasingly resistant to commonly used antibiotics such as ampicillin, ciprofloxacin, and trimethoprim-sulfamethoxazole (*14*, *15*). The current prevalence of recurrent UTIs, combined with the increasing loss of antibiotic efficacy, suggests that a more effective form of UTI prevention is a significant and urgent unmet need.

A vaccine that raises protective, long-term antibody responses against UTI-causing bacteria has the potential to meet this need. However, such a vaccine does not currently exist, and there are significant challenges to its development. Now, immunomodulating therapies are being explored for the treatment and prevention of recurrent UTIs, including orally delivered bacterial lysates of UPEC strains such as the commercially available OM-89 or sublingually delivered inactivated bacterial strains such as Uromune, which has reported results in humans (*16*, *17*). However, these approaches require extended dosing regimens (typically at least 3 months of daily dosing) and have not been shown to elicit long-lasting protection (*18*). In 2022, the sublingual inactivated bacteria vaccine candidate MV140 showed reduced UTI incidence at 9 months after either a 3- or 6-month period of intervention (*19*). The phase 1b trial of EXPEC4V, an intramuscular vaccine targeting the lipopolysaccharide-linked O-antigen of extraintestinal *E. coli*, showed no significant differences in UTI incidence (*20*).

As evidenced by these clinical results, generating safe, effective, and long-lasting immune responses across populations for UTI prevention is a major challenge. We sought to develop a novel strategy for UTI prevention to address this need. An ideal vaccine candidate would elicit immune responses that are specific to UTI-causing bacteria to avoid adverse effects to the microbiota while also targeting a broad range of UTI-causing pathogens. Furthermore, it is thought that both systemic responses in the blood and mucosal responses in the urogenital tract are important for protection against UTIs (*21*, *22*). These considerations suggest that an ideal vaccine for preventing UTIs would feature an ability to raise simultaneous responses against multiple highly specific and carefully selected epitopes targeting only pathogenic bacteria, an ability to elicit mucosal responses, and efficient dosing regimens that facilitate compliance and minimize cost.

To design a vaccine meeting these challenging criteria, we used a supramolecular approach to assemble multiple selected B cell epitopes from UPEC into sublingually immunogenic nanomaterials. Sublingual immunization is known to elicit antibody responses in the urogenital tract (*23*), but it is difficult to raise robust immune responses against short peptide epitopes via this route, because peptides are poorly immunogenic via the oral mucosa. Recently, we demonstrated with model epitopes that supramolecular peptide nanofibers bearing polymer modifications modulating mucus adhesivity are capable of raising strong systemic and mucosal antibody responses (*24*, *25*). This platform is based on extremely multivalent peptide nanofibers bearing muco-inert modifications such as short polyethylene glycol (PEG) chains or Pro-Ala-Ser peptides, and they raise antibody responses persisting for at least a year (*24*). The process of supramolecular assembly allows for the coassembly of peptide polymers bearing multiple selected pathogen-specific epitopes into integrated multiepitope nanofibers (*26*).

Here, we capitalize on the unique advantages of this platform to report a vaccine to prevent UTIs caused by UPEC. In mice, this vaccine elicited robust anti-UPEC antibodies that were not cross-reactive against commensal *E. coli*. Furthermore, the vaccines were as effective as high-dose oral antibiotics at protecting mice from lethal challenge with UPEC. We analyzed the composition of the gut microbiota and found that the nanofiber vaccine caused significantly less perturbation than antibiotics. In summary, we report a novel vaccination strategy, enabled by biomaterial design, that provides long-lasting, antibiotic-level efficacy against UPEC.

## RESULTS

### Selection of B cell epitopes from UPEC

Human UTIs can be caused by several genera of bacteria, but more than 80% are the result of infection by a diverse set of UPEC strains, collectively referred to as UPEC. While a vaccine against UPEC would not be universally effective in the human population, it would represent a marked step toward changing how UTIs are prevented and treated. UPEC themselves are a heterogeneous set of strains with variable expression of virulence factors such that a vaccine would still likely need to target multiple antigens to be broadly effective against UPEC alone (*1*). For these reasons, we sought to design a multivalent vaccine that would broadly target UPEC strains to prevent a large majority of UTIs.

**Fig. 1.**
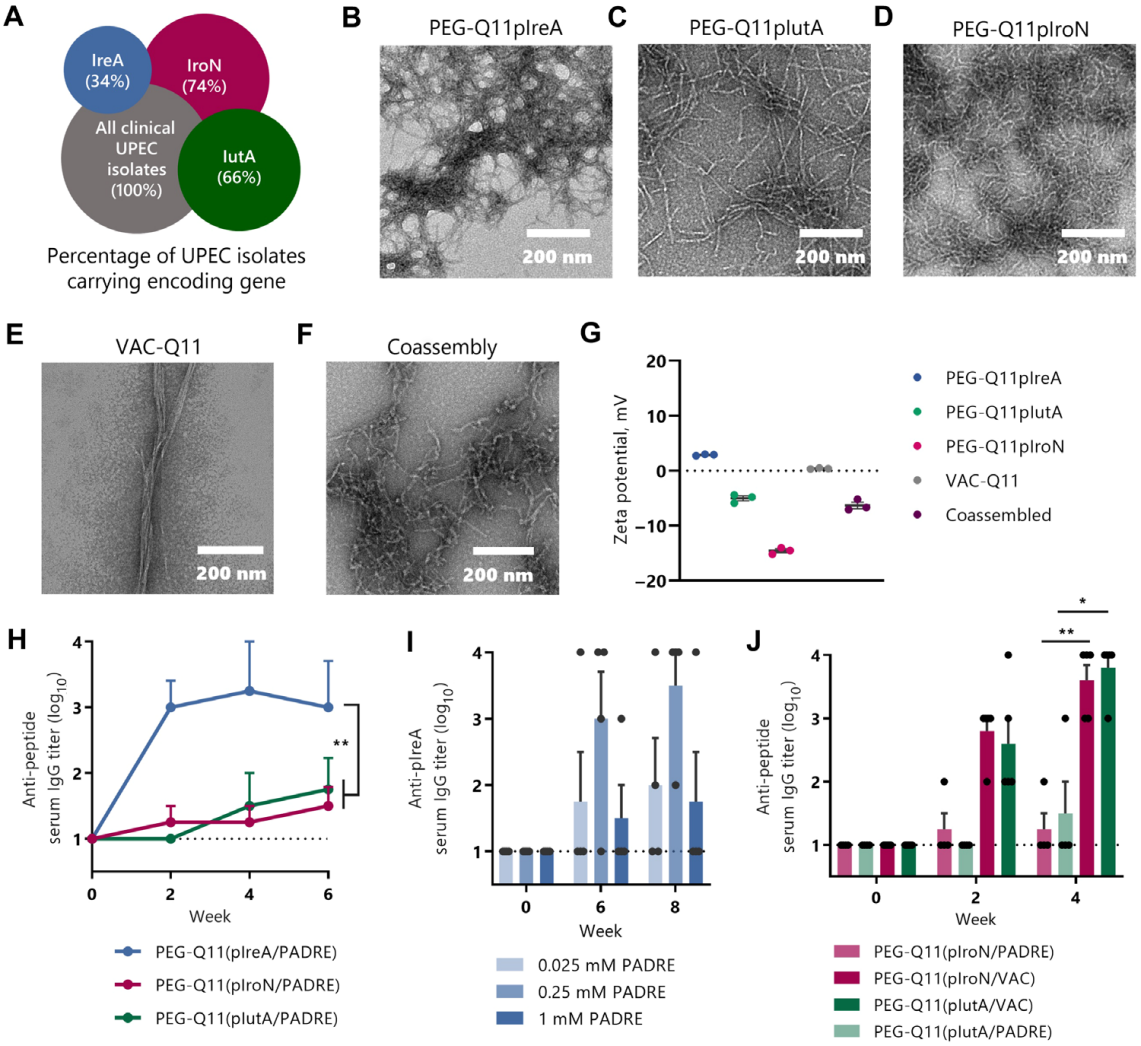
Sublingual nanofiber vaccine raises antibody responses against three B cell epitopes with broad expression across UPEC strains. (**A**) Percentage of clinical UPEC isolates that contain the gene encoding the parent proteins of the pIroN, pIutA, and pIreA epitopes (*2*). Transmission electron microscopy images of nanofibers composed of (**B**) PEG-Q11pIreA, (**C**) PEG-Q11pIutA, (**D**) PEG-Q11pIroN, and (**E**) VAC-Q11, or (**F**) a coassembly of these four together. (**G**) Zeta potential measurements of nanofiber assemblies. (**H**) Mice were immunized sublingually with coassembled PEG-Q11 nanofibers containing the PADRE T helper epitope and either pIreA, pIroN, or pIutA B cell epitope, plus cholera toxin B (CTB) adjuvant. Mice were boosted at weeks 1 and 3, and serum immunoglobulin G (IgG) titer against the immunizing epitope was measured [two-way repeated-measures analysis of variance (RM-ANOVA), Tukey’s test, *n* = 4 per group]. (**I**) Effects of titrating T cell epitope content with sublingual nanofiber vaccines. Mice were immunized sublingually with PEG-Q11(pIreA/PADRE) nanofibers containing variable concentrations of PADRE plus CTB and were boosted at weeks 1, 3, and 6 (*n* = 4 per group). (**J**) Coassembly with the VAC epitope, but not PADRE, elicits antibody responses against pIutA and pIroN. Mice immunized with the pIutA or pIroN epitope and VAC are compared to responses with PADRE shown in (F). All mice were boosted at weeks 1 and 3, and formulations contained CTB adjuvant (two-way RM-ANOVA, Tukey’s test, *n* = 4 per group).

To prioritize safety and avoid adverse effects related to off-target immune responses against nonpathogenic *E. coli* or other commensals, we sought peptide epitopes that would be found near-exclusively on UPEC. A major set of UPEC virulence factors is iron receptor proteins, which allow UPEC to survive in the iron-poor urinary tract (*1*). These receptors are ideal antigenic targets because, in addition to being critical to the survival of UPEC, they are surface-expressed, allowing for possible antibody binding. We selected three peptide epitopes previously identified within UPEC receptor proteins (*21*, *27*), one each from the proteins IreA, IutA, and IroN. The genes encoding these proteins were found in 34, 66, and 74% of clinical UPEC isolates (*2*), respectively, suggesting that, by targeting all three, we could design a vaccine that would be broadly effective against human infection with UPEC (Fig. 1A). Notably, the peptide epitopes from IutA and IroN have previously shown poor immunogenicity in mice after mucosal immunization, failing to raise systemic or urinary antibody responses even when given with high doses of the strong but nontranslatable cholera toxin adjuvant (*21*). We viewed these promising, yet poorly immunogenic, peptide epitopes as an opportunity to use our sublingual peptide immunization technology.

### Coassembly of T and B cell epitopes in sublingual nanofiber vaccines elicits antibody responses against UPEC epitopes

We sought to leverage our recently developed sublingual peptide vaccine platform (*24*) to enhance the immunogenicity of these challenging peptide epitopes and develop a highly specific anti-UPEC vaccine. We designed self-assembling peptide polymers with C-terminal peptide epitopes (referred to hereafter as pIreA, pIutA, and pIroN to distinguish them from their parent proteins), N-terminal PEG chains to promote sublingual mucus transport, and central Q11 peptide self-assembly domains to drive self-assembly into nanofibers (sequences in Table 1). To promote T cell help, we coassembled nanofibers containing both the UPEC B cell epitopes and either the universal helper T cell epitope PADRE (pan–human leukocyte antigen (HLA) DR–binding epitope) (*28*) or an alternate T helper epitope from vaccinia virus (referred to here as VAC) (*29*). Each of the constructs—PEG-Q11pIreA, PEG-Q11pIutA, PEG-Q11pIroN, and VAC-Q11, or a coassembly of all four—assembled into supramolecular nanofibers as evidenced by electron microscopy (Fig. 1, B to F). As reported previously (*24*, *30*), nanofibers were polydisperse with lengths ranging from under 100 nm to more than 1 μm. Zeta potential measurements of these fibers matched our previous characterizations (*25*, *30*), with PEG-conjugated nanofibers typically displaying slightly negative zeta potentials and non-PEGylated nanofibers being essentially neutral (Fig. 1G).

**Table 1.** Components of coassembled multivalent anti-UPEC vaccines.

**Name**	**Sequence**	**Function**
Q11	QQKFQFQFEQQ	Self-assembly into nanofibers
mPEG_2000_	CH_3_O-(CH_2_CH_2_O)*_n_* | Average *n* = 45	Mucus penetration
mPEG_2000_-pIroNQ11	mPEG_2000_-SGSGQQKFQFQFEQQSGSG- YLLYSKGNGCPKDITSGGCYLIGNKDLDPE-NH_2_	UPEC B cell epitope
mPEG_2000_-pIutAQ11	mPEG_2000_-SGSGQQKFQFQFEQQSGSG- VDDIDYTQQQKIAAGKAISADAIPGGSVD-NH_2_	UPEC B cell epitope
mPEG_2000_-pIreAQ11	mPEG_2000_-SGSGQQKFQFQFEQQSGSG- GIAKAFRAPSIREVSPGFGTLTQGGASIMYGN-NH_2_	UPEC B cell epitope
PADREQ11	NH_2_-aKXVAAWTLKAaSGSGQQKFQFQFEQQ-NH_2_	T helper epitope
VACQ11	NH_2_-QLVFNSISARALKAYSGSGQQKFQFQFEQQ-NH_2_	T helper epitope

On the basis of our previous work highlighting the importance of the ratio of T to B cell epitopes in nanofiber vaccination, we titrated the dose of PADRE to select for the most effective formulation (*31*). Sublingual immunization with 8 μl droplets of solutions containing PEG-Q11(pIreA/PADRE) fibers and the adjuvant cholera toxin B (CTB; the nontoxic B subunit of cholera toxin) led to robust pIreA-specific serum antibody responses (Fig. 1H). Interleukin-4–positive (IL-4^+^) T cell responses against the PADRE epitope were also observed (fig. S1). Notably, the antibody responses were dependent on the ratio of B to T cell epitope within the nanofibers (Fig. 1I). Coassembly of PADRE did not promote antibody responses against either pIutA or pIroN, leading us to focus on VAC. Sublingual immunization with PEG-Q11(pIutA/VAC) and PEG-Q11(pIroN/VAC) elicited epitope-specific antibody responses (Fig. 1J). The efficacy of the selected VAC epitope over PADRE in this instance could be due to differences in affinity of binding between the epitopes and major histocompatibility complex class II (MHC-II) molecules. The reported affinity of the VAC epitope for I-Ab (the haplotype of C57BL/6 mice) is 13.5 nM (*32*), compared with 94 nM for the PADRE epitope (*28*). These results highlight the ability of our vaccine platform to raise antibody responses sublingually against poorly immunogenic peptide epitopes, and the modularity of the materials made it straightforward to assess different types and amounts of T cell epitopes for advancement into the subsequent studies.

### Multivalent nanofibers raise simultaneous responses against selected UPEC B cell epitopes in serum and urine

**Fig. 2.**
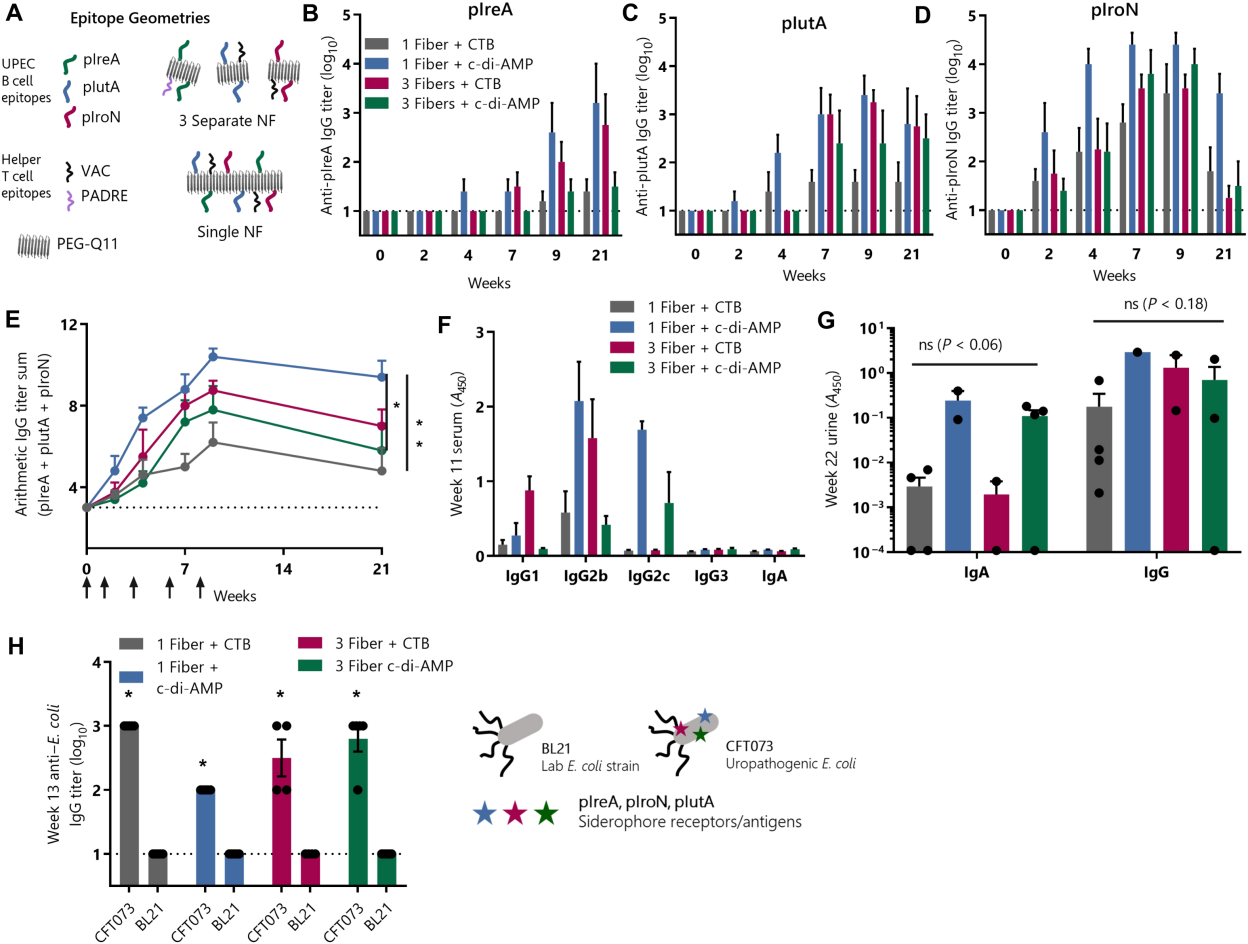
Fully coassembled nanofibers elicit polyvalent systemic and urinary antibody responses that specifically target UPEC. Mice were immunized sublingually with either a mixture of three separately assembled nanofibers [PEG-Q11(pIreA/PADRE) + PEG-Q11(pIutA/VAC) + PEG-Q11(pIroN/VAC)] or a single fully coassembled nanofiber [PEG-Q11(pIreA/pIutA/pIroN)] and either CTB or cyclic di-AMP (c-di-AMP) adjuvant and were boosted at weeks 1, 3, 6, and 8. (**A**) Schematic depicting the epitope composition of the single- and three-nanofiber (NF) formulations. (**B** to **D**) IgG levels were measured individually against each of the three peptide epitopes. (**E**) To compare the overall response, a titer sum was calculated by arithmetic addition of the titers against each of the three epitopes (two-way RM-ANOVA, Tukey’s test, *n* = 5 per group). (**F**) Serum antibody isotype and IgG subclasses were measured by enzyme-linked immunosorbent assay (ELISA) against a 1:1:1 mixture of pIreA, pIutA, and pIroN. (**G**) Urinary antibody levels were determined by ELISA on undiluted urine samples (two-way ANOVA, *n* = 1 to 4 per group). (**H**) Vaccine-induced serum antibodies bound specifically to UPEC. Week 13 serum IgG titers were measured by ELISA against a UPEC strain (CFT073) or a nonpathogenic laboratory strain (BL21) (multiple *t* tests, Holm-Šídák correction, *n* = 4 to 5 per group).

Having established that the selected B cell epitopes were immunogenic in peptide nanofibers, we next sought to design a multiepitope vaccine to elicit simultaneous antibody responses against all three epitopes: pIreA, pIutA, and pIroN. Again, we took advantage of the modularity of self-assembled peptide vaccines, which enabled a straightforward investigation of how the epitope arrangement within the nanofibers affected antibody responses in the context of different adjuvants. We immunized one set of mice with self-assemblies where all peptides were coassembled into homogeneous nanofibers bearing all three B cell epitopes and one T cell epitope, termed PEG-Q11(pIreA/pIutA/pIroN/VAC) (Fig. 2A). For another set, we synthesized three types of nanofibers, each bearing only one of the B cell epitopes along with T cell epitopes, and we immunized mice with mixtures of these three nanofibers, termed [PEG-Q11(pIreA/PADRE) + PEG-Q11(pIutA/VAC) + PEG-Q11(pIroN/VAC)]. We further compared these two formulations when adjuvanted with either CTB adjuvant or the STING agonist cyclic di-AMP (adenosine monophosphate). Each of the four formulations elicited epitope-specific antibody responses against each of the three UPEC B cell epitopes (Fig. 2, B to D). As a measure of the overall strength of the response, we calculated an arithmetic sum of the log_10_ immunoglobulin G (IgG) endpoint titers against the three epitopes. By this combined measure, the single nanofiber bearing all three B cell epitopes and adjuvanted with cyclic di-AMP elicited the strongest responses (Fig. 2E). The IgG subclasses of these responses were predominantly IgG2b and IgG2c, subclasses that are, in many cases, the most potent for antibacterial immunity (Fig. 2F) (*33*–*35*). The simplicity of the single nanofiber formulation and favorable translational profile of cyclic di-AMP, along with its effectiveness at raising high-titer responses, led us to select this formulation for future studies.

A major advantage of using sublingual immunization as the route for a UTI vaccine is its demonstrated ability to raise immune responses in the urinary tract. To characterize the ability of our vaccines to raise antigen-specific IgG and IgA within the urinary tract, we assayed urine from immunized mice (Fig. 2G). Individual groups were not statistically different from each other, although comparisons of IgA responses against different formulations approached significance, so to test the impact of adjuvant and nanofiber composition separately, we combined groups and ran *t* tests on urinary antibody levels, using Holm-Šídák correction for multiple comparisons. Nanofiber composition was not significant for either IgG (*P* = 0.95) or IgA (*P* = 0.95) when CTB and cyclic di-AMP adjuvanted groups were combined. In contrast, we found that cyclic di-AMP produced significantly higher epitope-specific IgA levels in the urine than CTB when one nanofiber and three nanofiber groups were combined (*P* = 0.038), while IgG levels did not differ significantly (*P* = 0.38). These results indicated that nanofiber sublingual vaccines could raise responses not only systemically but also within the urinary tract.

### Vaccine-induced antibody responses are specific for UPEC

A key motivation for using peptide epitopes is that, despite their generally poor immunogenicity, they are highly specific and, in principle, can be selected to target pathogenic bacterial strains while sparing nonpathogenic strains. To test the specificity of the antibody response raised by our vaccine, we compared their level of binding to both a uropathogenic strain of *E. coli* (CFT073) and a nonpathogenic laboratory strain of *E. coli* (BL21). Serum antibodies from all four immunization groups bound to CFT073 but had no detectable levels of anti-BL21 antibodies (Fig. 2H). Note that the CFT073 culture conditions used for this enzyme-linked immunosorbent assay (ELISA) are expected to induce expression of pIutA, but not pIreA or pIroN (*2*). These results still highlight that the induced antibodies do not bind nonpathogenic *E. coli* and that antibodies raised by peptide nanofibers are able to bind to CFT073. Along with the demonstrated ability to raise multivalent responses and to elicit urinary antibodies, this specificity is a unique advantage.

### Combined adjuvancy of STING and TLR9 agonists enhances urinary antibody levels after sublingual nanofiber immunization

**Fig. 3.**
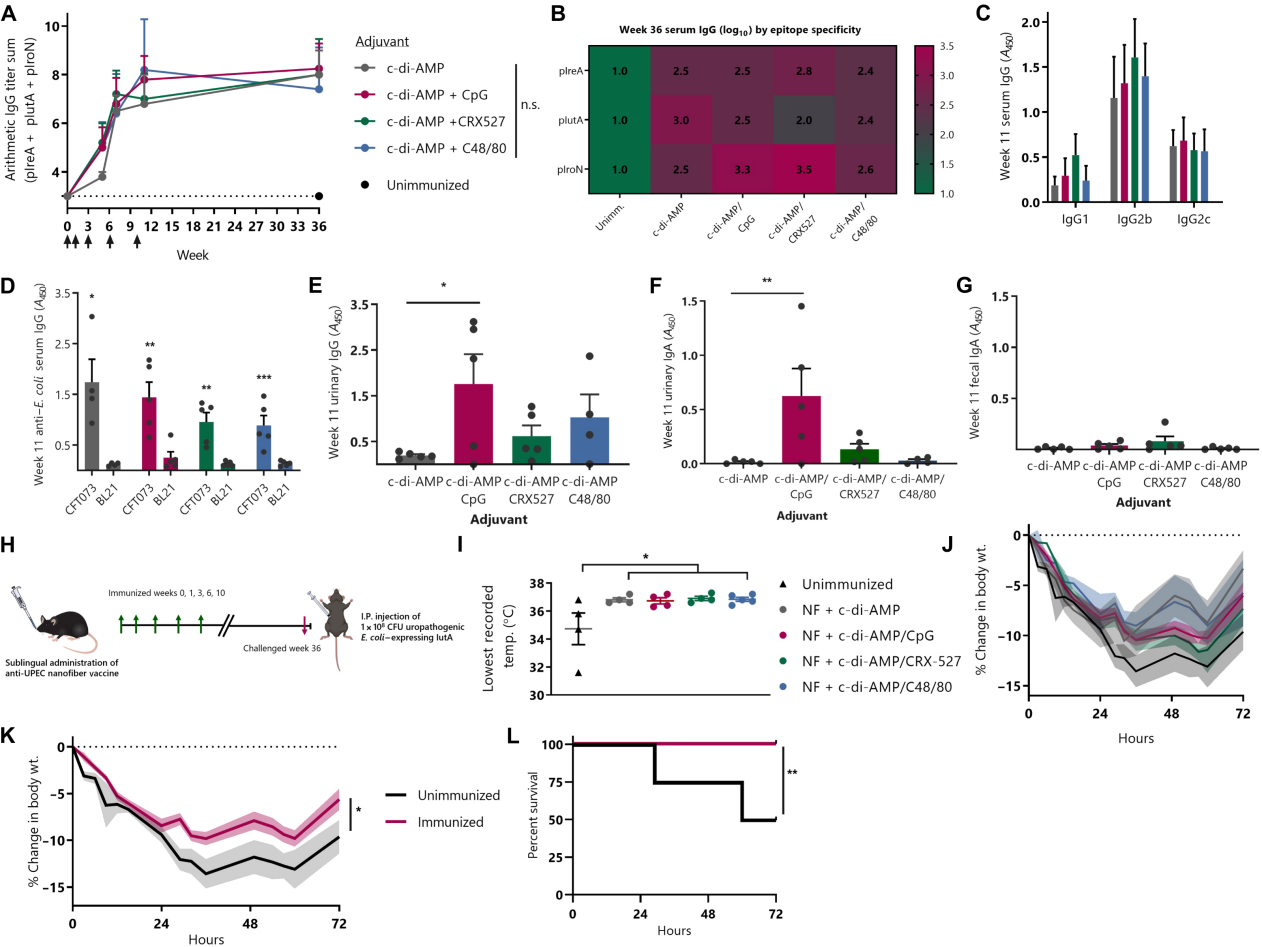
Formulation with STING and TLR9 agonists promotes strong serum and urinary antibodies against UPEC without accompanying gut responses. (**A**) Mice were immunized with PEG-Q11(pIreA/pIutA/pIroN/VAC) nanofibers and indicated adjuvants and boosted at weeks 1, 3, 6, and 10 (two-way RM-ANOVA, *n* = 5 per group). (**B**) Heatmap showing pIreA-, pIutA-, and pIroN-specific serum antibody responses at week 36. (**C**) IgG subclasses measured on serum diluted 1:1000 against 1:1:1 mixture of pIreA, pIutA, and pIroN. (**D**) Week 11 serum IgG measured by ELISA against UPEC strain (CFT073) or nonpathogenic laboratory strain (BL21) (multiple *t* tests, Holm-Šídák correction). (**E** and **F**) Urinary IgA and IgG at week 11 measured on undiluted urine against 1:1:1 mixture of pIreA, pIutA, and pIroN (one-way ANOVA, Dunnett’s multiple comparisons test against c-di-AMP only). (**G**) No antigen-specific fecal antibodies were observed after sublingual immunization. (**H**) Mice immunized in Fig. 3 (green arrows signify immunizations) were challenged intraperitoneally (I.P.) 26 weeks after final boost with 1 × 10^8^ CFU CFT073 cultured in Luria broth (pink arrow). (**I**) Lowest temperature recorded for each mouse (one-way ANOVA, Dunnett’s test versus unimmunized, *n* = 4 to 5 per group). (**J**) Body weight (wt.) over time. Bold lines represent mean of each group, and shaded boundaries indicate SEM. Weight and temperature curves for individual mice are in fig. S3. (**K**) Immunized groups were combined to show vaccine’s amelioration of weight loss versus unimmunized mice [two-way RM-ANOVA, *n* = 4 (unimmunized) or *n* = 17 (combined immunization groups)]. (**L**) Survival curve for unimmunized versus combined immunization groups (log-rank test).

On the basis of our initial vaccine studies, we proceeded with a formulation of a single coassembled nanofiber and cyclic di-AMP adjuvant. To enhance the urinary antibody responses elicited by our initial formulations, we examined the effects of combining cyclic di-AMP with additional adjuvants that act through different and potentially complementary pathways, such as those induced by Toll-like receptors (TLRs). Combinatory adjuvant approaches are well studied in the nanomaterial community (*36*) and have recently been applied in the context of UPEC vaccines (*37*). We sublingually immunized mice with PEG-Q11(pIreA/pIutA/pIroN/VAC) nanofibers and cyclic di-AMP, plus either CpG (a TLR9 agonist), CRX-527 (a TLR4 agonist), or C48/80 (a mast cell stimulator). The addition of these adjuvants did not have a significant effect on serum antibody responses, as all groups raised multivalent responses against all three B cell epitopes that were primarily of the IgG2b and IgG2c subclasses (Fig. 3, A to C). Again, these serum antibodies bound to pathogenic CFT073 *E. coli*, but not the laboratory strain BL21 (Fig. 3D). In contrast to the serum responses, however, the adjuvant combination strategy led to a significant increase in urinary antibodies. Specifically, the combination of cyclic di-AMP and CpG led to significantly greater levels of epitope-specific IgA and IgG in the urine (Fig. 3, E and F).

### Sublingual nanofiber vaccine raises no detectable gut IgA responses

An advantage of UPEC-specific vaccine strategies is that they avoid potential immune responses against commensal *E. coli* or other commensals. A further safeguard against such effects is a vaccine that raises antibody responses in the desired systemic and urinary compartments, but not in the microbe-rich gut. Sublingual vaccines have been shown in some cases to raise immune responses in the gastrointestinal tract (*38*–*40*). Swallowing of vaccine material could contribute to gut responses; notably, we use a conservative volume of 8 μl for our sublingual immunizations, well below the amount at which inadvertent swallowing would be expected (*41*). We tested whether the sublingual nanofiber vaccines were eliciting gut antibodies along with those observed in the blood and urine. In contrast to the observable levels of IgA, we did not detect any epitope-specific IgA in fecal samples of immunized mice (Fig. 3G). To confirm that our methods could detect fecal IgA, we examined the total fecal IgA and found it to be similar to published levels for C57BL/6 mice (fig. S2) (*42*).

### Sublingual vaccine protects against UPEC-mediated sepsis even with expression of a single antigen target

To test the clinical relevance of the antibodies raised by our sublingual vaccine, we challenged mice with CFT073, a UPEC strain isolated from a pyelonephritis patient (*43*). To directly assay the effectiveness of raised antibodies against the human pathogen, we performed intraperitoneal challenge of the previously immunized mice discussed above (Fig. 3H). This challenge also provides a model of UPEC-mediated sepsis; notably, urosepsis accounts for a quarter of all sepsis cases (*44*). The motivation for a multivalent vaccine strategy is to generate an immune response that is protective against UPEC with varying virulence factor expression profiles. To test whether expression of a single antigenic target on the infecting pathogen was sufficient for the nanofiber vaccine to afford protection, we cultured CFT073 under conditions in which the IutA protein, but neither the IreA nor IroN proteins, is expressed (*2*).

After challenging mice with CFT073, we monitored body temperature and weight loss for 72 hours to see whether vaccinated groups would ameliorate these expected clinical symptoms of infection (*45*). Unimmunized control mice exhibited significant loss of body temperature after challenge, compared with vaccinated mice (Fig. 3I and fig. S3). Body weight showed more variance between different immunization groups (which differed by adjuvant), but all immunized groups lost less weight compared with unimmunized mice throughout the study (Fig. 3J and fig. S3). To compare the overall effect of immunization in protecting against CFT073 challenge, we combined the results from the four groups of immunized mice. Collectively, the immunized mice showed significantly less change in body weight than did unimmunized mice (Fig. 3K). Furthermore, half of the unimmunized mice died as a result of the challenge, while all vaccinated mice survived (Fig. 3L). These results together indicated that the sublingual nanofiber vaccine provided protection against challenge with a human-infecting UPEC strain expressing a single targeted antigen.

### An accessible tablet vaccine formulation raises protective immune responses against UPEC epitopes in mice

**Fig. 4.**
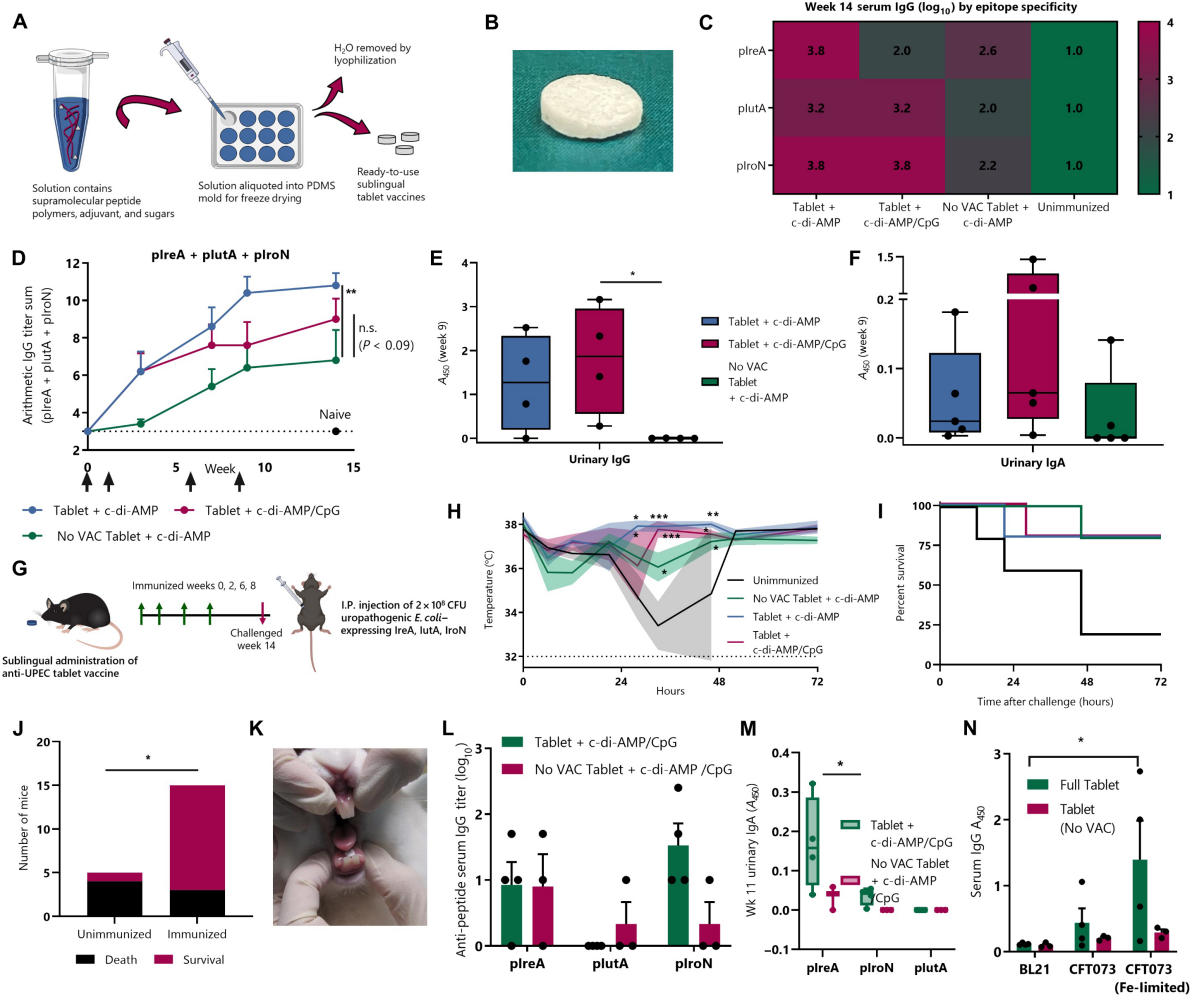
A highly accessible tablet delivery vehicle enables sublingual immunization against UPEC epitopes in mice and rabbits. (**A**) Schematic depicting tablet-making process. (**B**) Camera image of tablet vaccine (diameter, 5 mm). Mice were immunized with tablets containing PEG-Q11(pIreA/pIutA/pIroN/VAC) or PEG-Q11(pIreA/pIutA/pIroN; no VAC) and indicated adjuvants and boosted at weeks 1, 6, and 8. (**C**) Serum antibody responses against each UPEC epitope. (**D**) Serum antibody responses over time (two-way RM-ANOVA, Dunnett’s test against No VAC, *n* = 5 per group). (**E** and **F**) Urinary IgA and IgG in undiluted urine against 1:1:1 mixture of pIreA, pIutA, and pIroN (one-way ANOVA, Dunnett’s test against No VAC). (**G**) Experimental timeline. Immunizations are signified by green arrows; infection is signified by pink arrow. (**H**) Bold lines represent mean temperature, and shaded boundaries indicate SEM (two-way RM-ANOVA, Dunnett’s test against unimmunized, *n* = 5 per group). (**I**) Mice were sacrificed at humane endpoints or at 72 hours. Weight and temperature curves for individual mice are in fig. S4 (**J**) Contingency table depicting outcomes for unimmunized (*n* = 5) or immunized (*n* = 15) groups (Fisher’s exact test). (**K**) Camera image showing placement of tablet in rabbits. (**L**) Rabbits were immunized with vaccine tablets containing PEG-Q11(PIreA/PIutA/PIroN/VAC) or PEG-Q11(PIreA/IutA/IroN; No VAC), plus c-di-AMP and CpG adjuvants, and boosted at weeks 2, 4, 7, and 10 (*n* = 3 to 4 per group). (**M**) Urinary antibody responses in rabbits using undiluted urine against 1:1:1 mixture of pIreA, pIutA, and pIroN (two-way ANOVA, Šidák’s test). (**N**) Week 11 serum IgG against nonpathogenic BL21 *E. coli* or UPEC strain CFT073 cultured under normal or iron-limited conditions (two-way ANOVA, Tukey’s test).

Attitudes and perceptions regarding UTIs and resource limitations put constraints on the potential distribution of an anti-UPEC vaccine, particularly outside of wealthy nations. To achieve its most significant impact, a UTI vaccine would likely need to be cost-effective, suggesting the need for heat stability (to obviate expensive cold-chain storage) and simple administration procedures requiring minimal training. We recently developed a procedure for producing tablet vaccines based on self-assembling peptide-polymer nanofibers, which we termed SIMPL (supramolecular immunization with peptides sublingually) (*46*). SIMPL tablets are produced by lyophilization of solutions containing supramolecular nanofibers, sugar excipients, and adjuvant (Fig. 4A).

To test the effectiveness of a tablet formulation of an anti-UPEC nanofiber vaccine, we formed tablets containing PEG-Q11(pIreA/pIutA/pIroN/VAC) (Fig. 4B). We included either cyclic di-AMP adjuvant alone or both cyclic di-AMP and CpG, based on our finding that this adjuvant combination could enhance urinary antibody responses (Fig. 3). As a control, we also produced tablets containing PEG-Q11(pIreA/pIutA/pIroN) fibers, which lack the helper T cell epitope VAC (VACQ11 was replaced with Q11 in the formulation to keep total peptide concentration constant). Sublingual immunization with vaccine tablets performed similarly to the previously used droplet vaccines, raising multivalent responses against all three UPEC B cell epitopes (Fig. 4C). Among tablets with only the cyclic di-AMP adjuvant, tablets without the VAC epitope raised significantly lower serum antibody responses than those containing the T cell epitope (Fig. 4D).

Tablets without VAC did still raise detectable antibody responses against each of the epitopes, indicating that one of pIreA, pIutA, or pIroN may contain a T cell epitope within its sequence. Putative epitopes within the pIreA sequence have been reported for BALB/c mice (H-2-IAd haplotype) (*27*), although to our knowledge T cell epitopes within these peptides have not been reported for C57BL/6 mice. An in silico prediction that we performed using the consensus method (*47*, *48*) in the Immune Epitope Database and Analysis Resource did show potentially strong binding sequences to H-2-IAb within the pIreA sequence, which may, in part, account for the responses seen here.

The highest level of urinary IgG was observed in mice receiving tablets with the VAC epitope and both cyclic di-AMP and CpG adjuvants, again indicating that adjuvant combinations may be particularly important for stimulation of mucosal responses after sublingual nanofiber immunization (Fig. 4E). There was no detectable urinary IgG in mice that received tablets without the VAC epitope. Urinary IgA levels were not significantly different between groups but trended highest for the adjuvant combination group (Fig. 4F).

We next tested the protective ability of immune responses elicited by anti-UPEC SIMPL tablets. We challenged mice with a higher-lethality dose of 2 × 10^8^ colony-forming units (CFU) of CFT073, culturing these bacteria under iron-limited conditions that promote expression of IreA, IutA, and IroN proteins (Fig. 4G) (*2*). Nanofiber tablet vaccines were highly protective even against this increased dose, significantly ameliorating temperature drops and increasing survival (Fig. 4, H to J, and fig. S4). Immunization with any of the three tablet formulations led to 80% survival, compared with just 20% in unimmunized control groups.

### Anti-UPEC sublingual tablet vaccine raises epitope-specific serum and urinary antibodies in rabbits

To further demonstrate the translational potential of anti-UPEC nanofiber vaccines, we sought to use a more representative higher animal model. Compared with mice, rabbits have an oral cavity that is more similar to that of humans. The sublingual epithelium is nonkeratinized in rabbits and humans (but keratinized in mice) and contains greater numbers of cell layers in both rabbits and humans than in mice (*49*). These differences make rabbits a more desirable model for assessing the potential clinical effectiveness of a sublingually delivered vaccine. Immunization of rabbits with SIMPL tablets containing PEG-Q11(pIreA/pIutA/pIroN/VAC) or PEG-Q11(pIreA/pIutA/pIroN; no VAC) nanofibers and both cyclic di-AMP and CpG adjuvants elicited epitope-specific antibody responses (Fig. 4, K and L). Notably, the tablet immunizations were also capable of eliciting epitope-specific IgA responses in urine (Fig. 4M), and the antibodies bound most strongly to CFT073 cultured under iron-limiting uropathogenic conditions (Fig. 4N). Last, to observe the dissolution behavior of the tablets in a simulated clinical setting, we simulated the human oral cavity using 1.0 ml of pooled human saliva heated to body temperature (37°C) (fig. S5). Tablets rapidly dissolved under these conditions, with a median time of 20 s. Collectively, these results indicate the potential feasibility of progressing sublingual tablet vaccines toward clinical translation.

### Sublingual nanofiber vaccine is as effective at preventing UTI as high-dose oral antibiotics

**Fig. 5.**
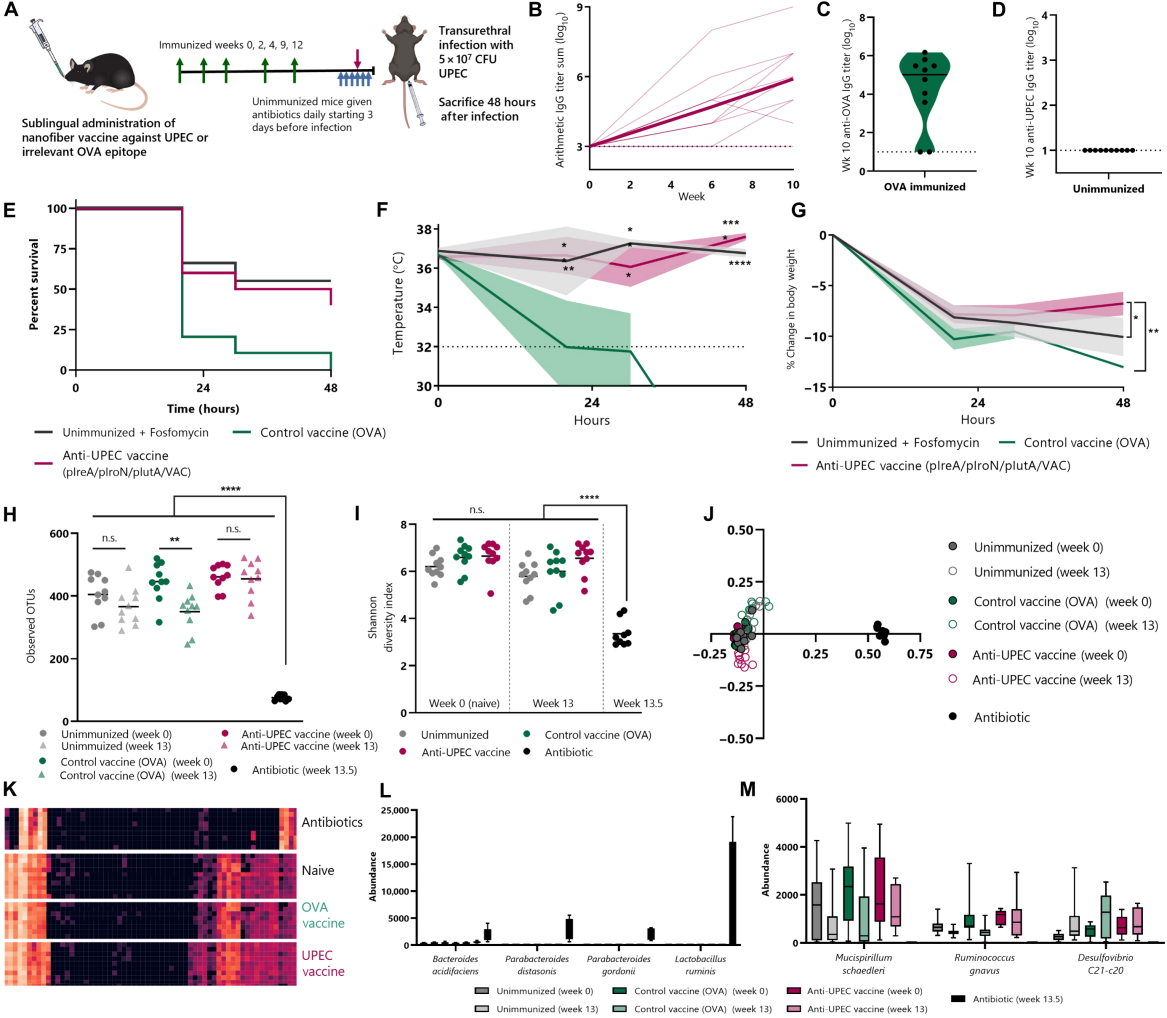
Sublingual anti-UPEC vaccine is as effective as high-dose antibiotics at preventing transurethral infection, but without accompanying disruption of the microbiome. (**A**) Mice (*n* = 10 per group) were immunized sublingually with PEG-Q11(pIreA/pIroN/pIutA) plus c-di-AMP and CpG and boosted at weeks 2, 4, 9, and 12 (green arrows). Control groups were unimmunized or immunized against irrelevant T and B cell epitope (OVA). After transurethral infection with 5 × 10^7^ CFU CFT073 (pink arrow), mice were sacrificed at 48 hours or humane endpoints. Unimmunized mice were given oral antibiotics (fosfomycin) at 1000 mg/kg daily for 3 days before and during infection (blue arrows). (**B**) Serum antibody responses in anti-UPEC vaccine group. Bold line shows mean titer, and faded lines represent individual mice. Titers against pIreA, pIroN, and pIutA are in fig. S6. (**C**) Serum IgG against OVA in mice given control vaccine. (**D**) Unimmunized mice raised no antibody responses. (**E**) Survival curve. (**F**) Mouse temperatures over time (two-way RM-ANOVA, Dunnett’s test against OVA vaccine). Bold lines show mean, and shaded areas represent SEM. (**G**) Body weight over time (two-way RM-ANOVA against OVA vaccine). Weight and temperature for individual mice are in fig. S7. (**H** to **M**) Feces collected before treatment (week 0) and week 13 for all groups plus week 13.5 for unimmunized groups (after 3 days of antibiotics) were used to compare effects of vaccination and antibiotics on microbiome. Richness sampling in fig. S8. (H) Estimation of microbiome’s OTU richness. (I) α-Diversity (one-way ANOVA, Tukey’s test). (J) Unweighted UniFrac principal coordinates analysis plot using β-diversity. (K) Family-level diversity. Rows are individual mice at week 13 (after vaccination) or week 13.5 (after antibiotic treatment). Full heatmap in fig. S9. (L) Overrepresented and (M) underrepresented species in antibiotic-treated mouse’s microbiome at the species level.

To place the efficacy of the anti-UPEC nanofiber vaccine in context, we compared it with antibiotics, the current gold standard of treatment for UTIs. We immunized mice with cyclic di-AMP and CpG adjuvants and either PEG-Q11(pIreA/pIutA/pIroN/VAC) nanofibers or, as a control, PEG-Q11OVA nanofibers. OVA_323–339_ is a model epitope that we have previously used in Q11-based nanofibers for raising sublingual B and T cell responses (Fig. 5A) (*24*, *46*). As expected, mice given the anti-UPEC vaccine raised antibody responses against the pIreA, pIutA, and pIroN epitopes while unimmunized mice did not, and ovalbumin (OVA)–immunized mice raised antibody responses against the OVA_323–339_ epitope (Fig. 5, B to D, and fig. S6). We challenged immunized mice transurethrally with 5 × 10^7^ CFU CFT073 to model the route of UTI and compared the results with unimmunized mice given repeated, high, daily doses of the oral antibiotic fosfomycin (Fig. 5A). While no mice given the control OVA vaccine survived to 48 hours after challenge, the vaccine performed similarly to the antibiotic treatment group (Fig. 5E). Notably, both the fosfomycin-treated and anti-UPEC–immunized groups maintained significantly higher temperatures than mice given the control vaccine (Fig. 5F and fig. S8). Notably, mice given the nanofiber vaccine had significantly less body weight loss than mice treated with fosfomycin (Fig. 5G and fig. S8). The lack of efficacy of the control vaccine against OVA demonstrated that the effect of the anti-UPEC nanofiber vaccine was attributable to its antigen specificity for CFT073 and not nonspecific effects due to adjuvants or the nanofibers themselves.

### Anti-UPEC vaccine is minimally disruptive of the microbiome

While antibiotics are highly effective at treating UTIs, there are concerns about their long-term prophylactic usage due to effects on the patient’s microbiome. We sought to demonstrate that our vaccine strategy would allow for long-term protective responses that were minimally disruptive of the microbiome due to their specificity for UPEC. We collected feces from unimmunized, anti-UPEC–immunized, or anti-OVA–immunized mice before (week 0) and after (week 13) treatment. We also collected feces from the unimmunized group after three daily doses of fosfomycin, before transurethral challenge (week 13.5).

The difference between the microbiome composition of antibiotic-treated mice and all other groups was stark. The OTU (operational taxonomic unit) richness of the microbiome was markedly reduced after antibiotic treatment, while immunization with the anti-UPEC nanofiber vaccine had no effect (Fig. 5H). Furthermore, while the Shannon diversity index (a measure of α-diversity) was statistically identical between all non–antibiotic-treated groups, the antibiotic group was highly significantly different from these groups (Fig. 5I). To highlight this, we used multidimensional scaling to plot the similarity of each mouse (Fig. 5J). The antibiotic mice formed a distinct cluster apart from all other mice. To further probe the differences, we generated a heatmap displaying the relative abundance of microbiome members at the family taxonomic level (Fig. 5K). Antibiotic-treated mice had a shift in microbiome composition that included increased levels of the species *Bacteroides acidifaciens*, *Parabacteroides distasonis*, *Parabacteroides gordonii*, and *Lactobacillus ruminis*, in addition to reduced levels of the species *Mucispirillum schaedleri*, *Ruminococcus gnavus*, and *Desulfovibrio C21-c20* (Fig. 5, L and M). These results support the possibility of sublingual vaccines as a safe, long-term prophylactic approach to preventing UTIs.

## DISCUSSION

In this study, we report the design of an anti-UPEC vaccine that leverages the unique advantages of a sublingual supramolecular nanofiber platform. A single highly coassembled nanofiber containing a helper T cell epitope raised simultaneous antibody responses against three UPEC peptide epitopes. Mice immunized with optimized formulations were protected from both systemic and transurethral UPEC challenge and induced minimal changes in the mouse microbiome.

This study presents a strategy with several advantages for progression toward clinical translation. Our vaccine strategy prioritizes safety, a critical consideration for a viable UTI vaccine. The threshold for safety is high for the design of a vaccine against a generally non–life-threatening disease. Here, we show the ability to raise systemic and mucosal responses against peptide UPEC epitopes that have previously been identified as ideal antigenic targets but suffered from poor immunogenicity. Two of these epitopes have previously been shown to raise no detectable responses even when adjuvanted with the strong but nontranslatable full cholera toxin (*21*). In stark contrast, our vaccine raised not only systemic titers but also epitope-specific antibody responses in urine while using the more favorable nucleotide adjuvants cyclic di-AMP and CpG. Both classes of adjuvants, cyclic dinucleotides (CDNs; such as AMP) and oligodeoxynucleotides (like CpG), have shown translational promise in clinical trials (*50*, *51*). Because of the low immunogenicity of these targets, our immunization schedule included a prime and four boosters to promote durable antibody responses against all three UPEC epitopes. Further optimizations to dose and booster spacing may reduce the need for boosters and are important considerations for translation.

Another translational consideration is the potential for misadministration in humans, leading to swallowing of vaccine material; however, we consider this risk minimal for several reasons. First, our tablets dissolve rapidly (fig. S5), which reduces the potential for oral delivery rather than sublingual. Second, we have previously shown that our sublingual nanofiber vaccines have prolonged residence up to 12 hours at the administration site, despite ad libitum access to food and water, indicative of retention at the sublingual epithelium. Last, studies with similar self-assembling nanofiber vaccines have shown that these materials are rapidly degraded in the harsh gut environment, inhibiting their immunogenicity (*52*).

Because of the lack of an established UTI vaccine raising long-term responses in humans, some consequences of such a vaccine are not fully known. Particularly critical is the possibility of a nonspecific vaccine that, for example, raises responses that target both UPEC and nonpathogenic *E. coli*. Approaches that are based on the use of inactivated pathogens or lyophilized microbial proteins may contain epitopes that do not discriminate between pathogenic and nonpathogenic microbes. Given the importance of the microbiota and the damage that can result from continuous use of antibiotics, long-lasting off-target immune responses could have detrimental effects. We show that nanofiber-generated antibodies bind to a UPEC strain and induce protection without showing any cross-reactivity toward a nonpathogenic *E. coli* strain. As an additional safeguard, our vaccine raises responses in the urinary tract and blood without eliciting detectable IgA antibodies in the feces, suggesting that there is minimal response in the commensal microbe-rich gastrointestinal tract. Most notably, our vaccine did not induce notable broad changes in the microbiome of mice. In stark contrast, mice treated with the oral antibiotic fosfomycin had significantly altered microbiome composition. The importance of our vaccine’s ability to raise protective responses without disrupting the microbiome is heightened by evidence suggesting that microbiome changes can be passed down generationally (*53*, *54*).

The sublingual route not only affords the ability to raise systemic and urinary titers but also provides key logistical benefits. Our tablet formulation has been shown to be heat stable (*46*), which may reduce or eliminate the high costs and infrastructure required for cold-chain distribution of a vaccine and enable a far greater global distribution compared to vaccines that are dependent on refrigeration. The limitations of cold chain–dependent vaccine distribution have been acute and prominent during the severe acute respiratory syndrome coronavirus 2 (SARS-CoV-2) pandemic, heightening awareness of this critical aspect of vaccine development. An additional advantage of sublingual tablet-based vaccination is the potential for self-administration, which could also help to facilitate its adoption. Our tablet vaccine raised responses not only in mice but also in rabbits, which have an oral cavity with key similarities to humans. Safety and efficacy studies in higher animal models are still needed, but collectively, the results presented in this study provide a strong initial indicator of a favorable efficacy and safety profile.

A key rationale for the use of the model UPEC strain CFT073 is that it was isolated from a human patient (*43*). This indicates that the epitope specificities of the antibodies that we raised are clinically significant. However, it remains to be determined how effective our vaccine is against other UPEC strains. Our multivalent strategy is designed so that most UPEC should express at least one of the three antigenic target proteins (IreA, IutA, and IroN). For CFT073, our results suggest that expression of a single target may be sufficient. Expression varies significantly across UPEC; however, testing our antibodies for binding against a panel of clinical isolates would give a greater indication of the predicted effects on a population level.

We focused on designing a vaccine against UPEC, which causes at least 80% of uncomplicated UTIs. The remaining 20% are caused by several genera of pathogens, including *Staphylococcus*, *Klebsiella*, *Proteus*, and *Pseudomonas* (*55*). Designing a “universal” UTI vaccine would present a challenge, particularly for a safety-focused approach such as this one that relies on peptide epitopes not shared with nonpathogenic microbes. In addition, polymorphism in human HLA could pose a challenge for a universal peptide-based vaccine in humans due to the requirement for T cell help. However, the modularity of our coassembled vaccines does present the opportunity to address these concerns through inclusion of additional pathogen-specific B cell epitopes and helper T cell epitopes. Polymorphisms in human STING could also limit broad effectiveness of adjuvanting with CDNs. While we focused on CDNs due to their more established status, 2′,3′-cGAMP has been shown to broadly activate human STING alleles and induce more potent activation than CDNs, and its use is an interesting consideration (*56*, *57*). A related question is whether our vaccine would be as effective in its target population of individuals with previous recurrent UTIs, because our infection models were performed by vaccinating mice before any infection. Further experiments are needed to answer this question, but we note that clinical trials of vaccines and immunotherapies for UTI are performed in individuals with histories of infection (*16*, *17*, *19*, *20*), and this is not cited as a barrier to a successful therapy.

In summary, we demonstrated the ability to raise anti-UPEC antibodies using a sublingual nanofiber vaccine platform. This vaccine generated antibodies against three unique targets from UPEC. These antibodies were found both systemically and in the urinary tract and bound to a model UPEC strain (CFT073) without showing any binding to a nonpathogenic laboratory strain of *E. coli*. In a UPEC-mediated sepsis model, vaccination ameliorated symptoms and enhanced survival. We further demonstrate the ability to formulate our anti-UPEC vaccine as a sublingual tablet, and that this formulation is effective in both mice and rabbits. Critically, our vaccine was as effective as antibiotics in a transurethral challenge with CFT073 while minimally disrupting the microbiome. This work represents an early step toward a safe and broadly effective vaccine to prevent UPEC-mediated infection, and it leverages the unique advantages of a sublingual supramolecular nanofiber vaccine platform to address this challenge.

## METHODS

### Peptide-polymer synthesis and nanofiber preparation

Peptides were synthesized using standard Fmoc solid-phase synthesis on Rink amide resins. PEG peptides were synthesized by on-resin conjugation of 2000 MW mPEG-NHS (Creative PEGWorks PLS-214) to the N terminus. Peptides were cleaved for 2 hours at room temperature in a 95/2.5/2.5 trifluoroacetic acid/triisopropylsilane/water cocktail, followed by washing with cold diethyl ether. Peptides were purified by reversed-phase high-performance liquid chromatography using a C4 column (PEG-peptides) or C18 column (non-PEGylated peptides) and lyophilized. Peptide identity was confirmed using matrix-assisted laser desorption/ionization (MALDI) mass spectrometry on a Bruker Autoflex Speed LRF MALDI-TOF spectrometer using α-cyano-4-hydroxycinnamic acid (Sigma-Aldrich, 70990) as the matrix.

To prepare nanofiber solutions, lyophilized peptides were dissolved at 8 mM in sterile water and incubated at 4°C overnight. The solutions were then brought to the final concentrations in 1× phosphate-buffered saline (PBS) by addition of sterile water and sterile 10× PBS and incubated at room temperature for 3 hours before use to allow for fibrillization. Coassembled nanofibers were prepared by vortexing lyophilized B cell epitope peptides (PEG-Q11pIreA, PEG-Q11pIutA, and PEG-Q11pIroN) for 20 min and dissolving in a VACQ11 or Q11 solution at 8 mM total peptide. For multiple nanofiber formulations for immunization, each nanofiber was separately assembled and combined just before immunization.

For adjuvanted solutions, nucleotide adjuvants (cyclic di-AMP and CpG) were added just before fibrillization; all other adjuvants were added after fibrillization. Adjuvants were included in formulations at the following dosages per mouse: 10 μg of CTB subunit (List Labs, 104), 10 μg of cyclic di-AMP (InvivoGen, vac-nacda), 25 μg of CpG (InvivoGen, tlrl-1826), 20 μg of CRX-527 (InvivoGen, tlrl-crx527), and 15 μg of compound 48/80 (MilliporeSigma, C2313). Adjuvant dosages for CTB and cyclic di-AMP were selected on the basis of our previously published work with sublingual nanofiber immunization (*24*, *25*, *46*). Doses for CpG, CRX-527, and C48/80 were selected on the basis of their use in the literature.

### Nanofiber characterization

To visualize nanofiber morphology by transmission electron microscopy, nanofiber solutions were diluted to 0.2 mM in 1× PBS and deposited onto Formvar/carbon-coated 400 mesh copper grids (Electron Microscopy Sciences, EMS400-Cu) for 1 min, rinsed with ultrapure water, and negatively stained for 1 min with 1% (w/v) uranyl acetate (EMS, 22400-1) before wicking away with filter paper. Samples were imaged on an FEI Tecnai G^2^ Twin electron microscope at 120 kV.

The zeta potential was measured on fibrillized nanofiber solutions diluted to 0.2 mM in 1× PBS. Solutions were analyzed at 25°C using Anton Paar Litesizer 500.

### Tablet production process

Reverse tablet molds were designed in FreeCAD and three-dimensionally printed with MakerBot Ultimaker 3. Polydimethylsiloxane (PDMS) molds were prepared using SYLGARD 184 kits (Sigma-Aldrich, 761028). Fibrillized nanofiber solutions were mixed with adjuvants and sugars to a final concentration of 7.8 weight % each of trehalose (Santa Cruz Biotechnology, 394303), dextran (Alfa Aesar, J61216), and mannitol (MilliporeSigma, M4125). Final solutions were pipetted into the PDMS tray (30 μl per tablet), frozen at −80°C, and lyophilized (*46*).

### Estimation of clinical tablet dissolution time

Five immunization-formulation tablets containing PEG-Q11(pIreA/pIutA/pIroN/VAC) were individually tested. For each tablet, 1.0 ml of pooled human saliva (Innovative Research, IRHUSL) was warmed to 37°C and dispersed on a petri dish. A tablet was dropped atop the saliva, and the dissolution time was measured, using USP (United States Pharmacopeia) guidelines to determine the point of disintegration (www.usp.org/sites/default/files/usp/document/harmonization/gen-chapter/april-2019-m99460.pdf). A previous study showed 1.0 ml to be the approximate volume of saliva in a human mouth before swallowing (*58*).

### Animals and immunizations

Animal experiments were approved by the Institutional Care and Use Committee of Duke University under protocol no. A264-18-11. Murine immunizations were initiated with female C57BL/6 mice (Envigo) aged 9 to 12 weeks. For sublingual immunizations, mice were deeply anesthetized by a cocktail delivering ketamine (100 mg/kg) and xylazine (10 mg/kg). For droplet immunizations, a micropipette with a 20-μl tip was used to apply 8 μl of the immunizing solution below the tongue. For tablet immunizations, the tablet was placed below the tongue of anesthetized mice using tweezers. For droplet and tablet immunizations, the mouse’s heads were placed in anteflexion for 20 min following administration to prevent swallowing of the material.

Rabbit immunizations were conducted with 8-month-old female New Zealand White rabbits (Envigo). Rabbits were anesthetized with ketamine (20 mg/kg) and xylazine (3 mg/kg). Tablets were placed under the rabbits’ tongue using tweezers, rabbits’ head were placed in anteflexion for 20 min, and recovery was initiated by atipamezole (xylazine reversal agent).

The total peptide concentration was 5 mM for all immunizations. For single-epitope immunizations with PADRE, the B cell epitope was 4.75 mM and PADRE was 0.25 mM, unless indicated otherwise. For single-epitope immunizations with VAC, the B cell epitope was 4.4 mM and VAC was 0.6 mM. For coassembled multi–B cell epitope nanofibers (including tablets in both mice and rabbits), each B cell epitope was 1.4 mM and VAC was 0.6 mM. For mixed nanofiber formulations, each B cell epitope was at three times the final concentration, to keep total epitope dose consistent with single nanofiber formulations after mixing. For no VAC tablets, VACQ11 was replaced with the same concentration of unmodified Q11 to keep the total peptide concentration constant.

### Measurement of antibody responses

Serum was collected via the submandibular vein. For analysis of epitope-specific IgG by ELISA, plates were coated with streptavidin (20 μg/ml; MilliporeSigma, 189730) overnight at 4°C. Plates were washed with Tween 20 (0.5 g/liter) in PBS (1× PBST), coated for 1 hour with biotinylated pIreA, pIutA, or pIroN (20 μg/ml), and blocked with Super Block Blocking Buffer (Thermo Fisher Scientific, 37515). When indicated in the figure legends, plates were coated with a 1:1:1 mixture of biotinylated pIreA, pIutA, and pIroN, at a total concentration of 20 μg/ml. Sera diluted in PBST–bovine serum albumin or undiluted urine were added to the plate, and antigen-specific IgG was detected by horseradish peroxidase (HRP)–conjugated Fcγ fragment–specific goat anti-mouse IgG (Jackson ImmunoResearch, 15-035-071). Results are reported as antibody titers calculated with an absorbance cutoff of 0.2 OD (optical density), or as background-subtracted *A*_450_ (absorbance at 450 nm) values. For measuring antibody subclasses, HRP-conjugated goat anti-mouse detection antibodies were purchased from SouthernBiotech (IgG1, 1071-05; IgG2b, 1091-05; IgG2c, 1078-05). For whole-cell ELISAs, bacteria were cultured as indicated in the following section, and plates were coated with an OD_600_ = 1.0 solution in 1× PBS overnight and blocked for 2 hours with 10% heat-inactivated fetal bovine serum in 1× PBS before addition of sera. Total (non–antigen-specific) IgA was determined using a mouse IgA ELISA kit (Thermo Fisher Scientific, 88-50450).

### Bacterial culture and challenge model

The UPEC strain CFT073 was purchased from the American Type Culture Collection (700928); this strain was originally isolated from a pyelonephritis patient (*43*). CFT073 was cultured at 37°C with aeration in LB (tryptone, 10 g/liter; yeast extract, 5 g/liter; sodium chloride, 10 g/liter) at a 1:100 dilution from a starter culture. Under iron-limited culture conditions, the chelator 2,2′-bipyridyl (MilliporeSigma, D216305) was present at 400 μM in LB. The UPEC strain BL21 was purchased from New England BioLabs (C2530H), and culture was performed as with CFT073 but with the use of terrific broth (Fisher Scientific, BP2468).

For the bacteremia model, immunized or naive mice were challenged with intraperitoneal injection of 1 × 10^8^ or 2 × 10^8^ CFU of CFT073 in 1× PBS, as specified in the figure captions. Temperature and body weight of mice were recorded at regular intervals for 72 hours. Humane endpoints were set as a temperature below 32°C, loss of 25% of body weight, or exhibition of significant clinical symptoms, as prescribed by the Institutional Animal Care and Use Committee guidelines.

For the transurethral infection model, water supply was removed from cages for 1 hour before infection to allow for voiding of bladder. Mice were sedated with an intraperitoneal injection of a ketamine/xylazine cocktail, and a sterilized plastic needle dabbed in surgical lubricant was used to deliver 5 × 10^7^ CFU of CFT073 into the urethra. Mice were monitored as for the bacteremia model but sacrificed at 48 hours.

### Microbiome analysis

Fecal pellets were collected from mice and immediately frozen on dry ice before storage at −80°C. Samples were submitted to the Duke Center for Genomic and Computational Biology Microbiome Shared Resource core for microbiome analyses. In brief, the Duke Microbiome Core Facility (MCF) extracted DNA using the Qiagen PowerSoilPro DNA Kit (Qiagen, 47014). Bacterial community composition in isolated DNA samples was characterized by amplification of the V4 variable region of the 16*S* ribosomal RNA (rRNA) gene by polymerase chain reaction (PCR) using the forward primer 515 and reverse primer 806 following the Earth Microbiome Project protocol (www.earthmicrobiome.org/). These primers (515F and 806R) carry unique barcodes that allow for multiplexed sequencing. Concentration of the PCR products were accessed using a Qubit dsDNA HS assay kit (Thermo Fisher Scientific, Q32854) and a Promega GloMax plate reader. Equimolar 16*S* rRNA PCR products from all samples were pooled before sequencing, which was performed by the Duke Sequencing and Genomic Technologies shared resource on an Illumina MiSeq instrument configured for 250–base pair (bp) paired-end sequencing runs.

Sequences were analyzed using QIIME2 software. Demultiplexed forward reads were assigned a cutoff between 10 and 230 bp, and reverse reads were assigned a cutoff between 10 and 160 bp. Samples were trimmed to a depth of 94,132 to retain 6,212,712 (73.67%) features in 66 (94.29%) samples. This sampling depth was used to rarefy the feature table to calculate α- and β-diversity via a phylogenic rooted tree. Alpha rarefaction was plotted to confirm that the richness of the samples had been fully observed. Sequences were then classified against the Greengenes 13_8 99% OTU full-length sequence training set for taxonomic analysis.

### Statistical analysis

Statistical analyses were performed using GraphPad Prism 9. Statistical tests used for each experiment are indicated in brackets in the figure captions. All values are reported as means ± SEM, with sample sizes indicated in the figure captions. Statistical significance is indicated in the figures as n.s. (nonsignificant), *P < 0.05, **P < 0.01, and ***P < 0.001.

## Notes

View/request a protocol for this paper from *Bio-protocol*.
