# Supplementary material for: A sublingual nanofiber vaccine to prevent urinary tract infections
Source: Sci Adv. 2022 Nov 23;8(47):eabq4120. doi: 10.1126/sciadv.abq4120 (PMC9683704; doi:10.1126/sciadv.abq4120)
Supplement: Supplementary file 1 — Figs. S1 to S9 [file sciadv.abq4120_sm.pdf]

Science Advances
AAAS

Supplementary Materials for

A sublingual nanofiber vaccine to prevent urinary tract infections

Sean H. Kelly *et al.*

Corresponding author: Joel H. Collier, joel.collier@duke.edu

Sci. Adv. **8**, eabq4120 (2022)
DOI: 10.1126/sciadv.abq4120

This PDF file includes:

Figs. S1 to S9

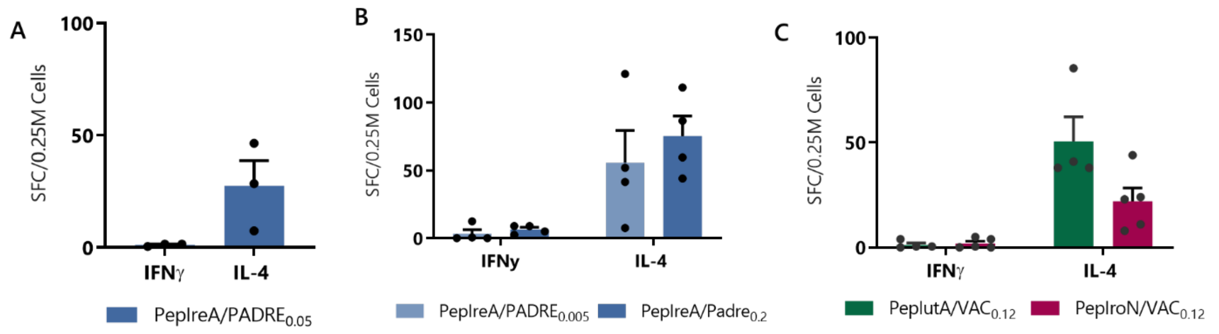

Figure S1*:* T-cell responses with sublingual immunization against individual B-cell/T-cell epitope co-assemblies. ELISPOT was performed on splenocytes harvested from mice immunized in Figure 1. Mice were boosted with the immunizing formulation one week before sacrifice. Cells were stimulated with the immunizing T-cell epitope. SFC: spot-forming cells. Subscripts indicate the molar fraction of the T-cell epitope within the co-assembled nanofiber. **(A-B)** Corresponds to mice immunized with PEG-Q11(pIreA/PADRE) at differing doses of PADRE in Figure 1I. Mice in A were sacrificed at week 73, mice in B were sacrificed at week 64. **(C)** Corresponds to mice immunized with PEG-Q11(pIroN/VAC) or PEG-Q11(pIutA/VAC) in Figure 1J, sacrificed at week 53.

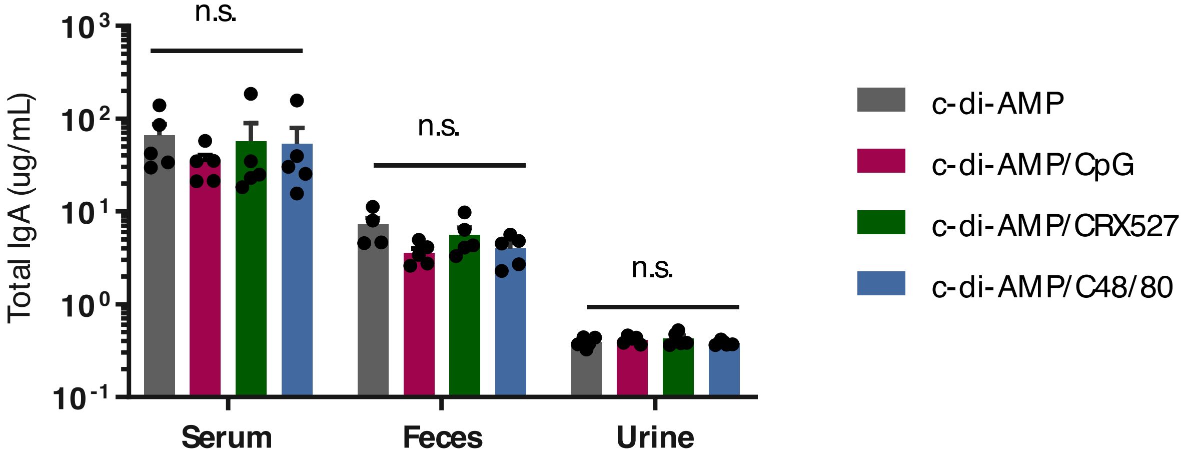

Figure S2*: Total (non-antigen specific) IgA levels in serum, fecal extracts, and urine.* Total IgA in sera, fecal extract, and urine were determined for mice immunized in Figure 3A using an ELISA kit. n.s. = non-significant by 2-way ANOVA with Tukey's multiple comparisons test, n=5/group.

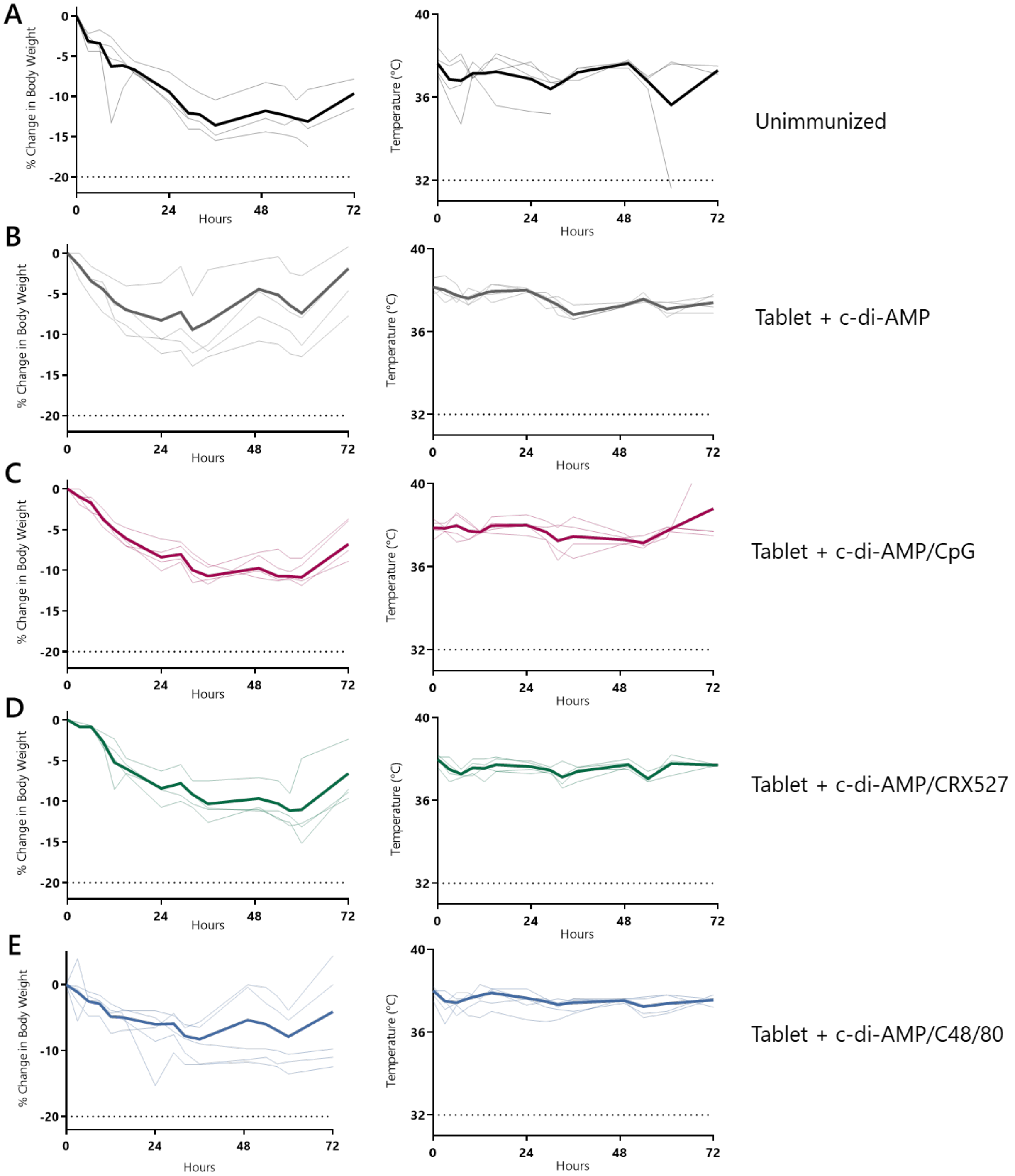

Figure S3: Body weight (left) and temperature (right) curves for individual mice in sepsis challenge from Figure 3H.

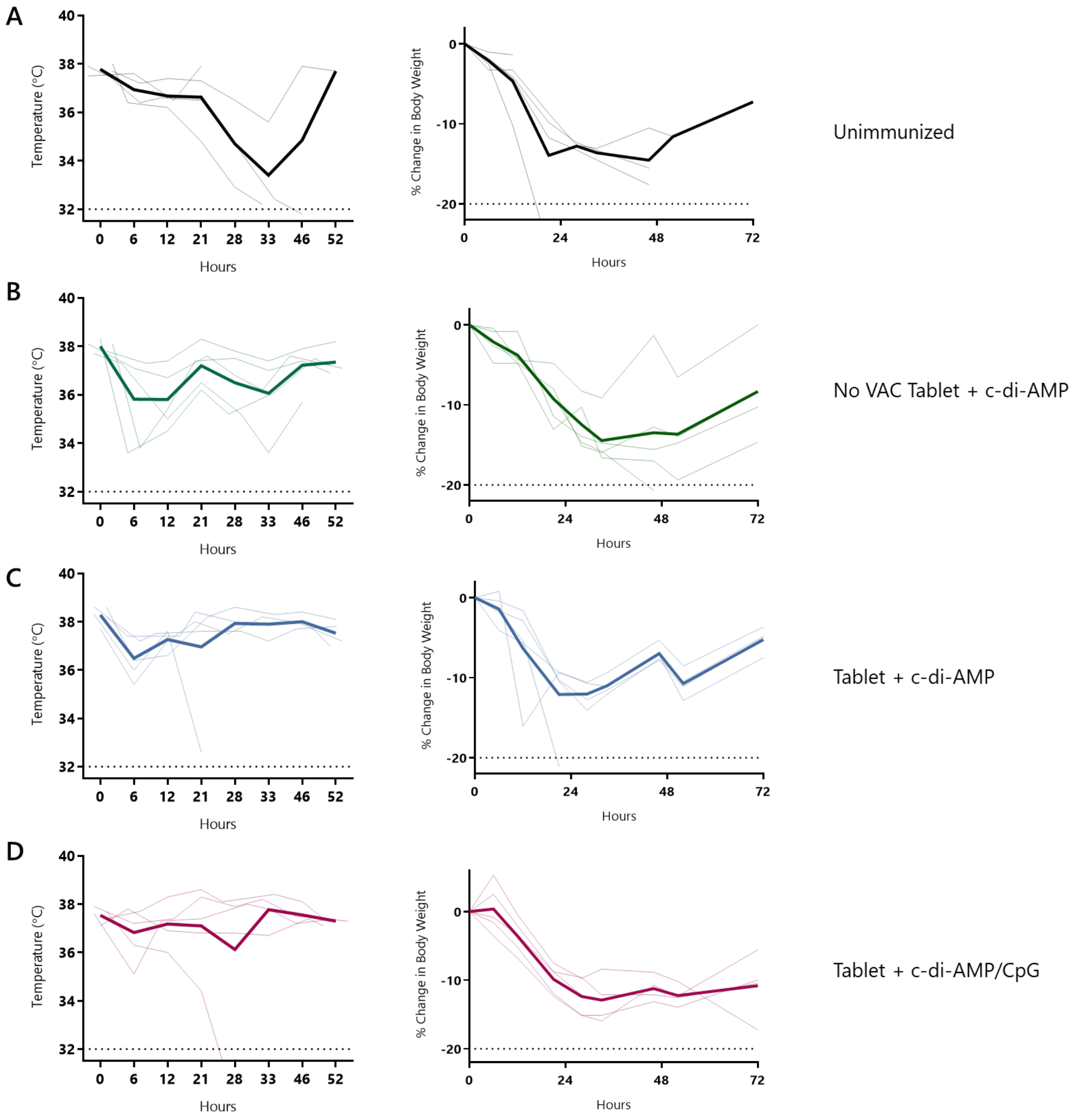

Figure S4: Body weight (left) and temperature (right) curves for individual mice in sepsis challenge in Figure 4G.

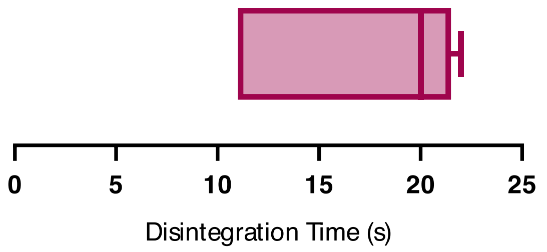

Figure S5: Simulation of tablet disintegration in human oral cavity. Immunization formulation UTI tablets containing PEG-Q11(pIreA/pIutA/pIroN/VAC) nanofibers and cyclic-di-AMP adjuvant were placed into 1 mL of human saliva heated to 37 ºC, and disintegration time was measured. Solid line at 20 seconds represents the median disintegration time. n = 5.

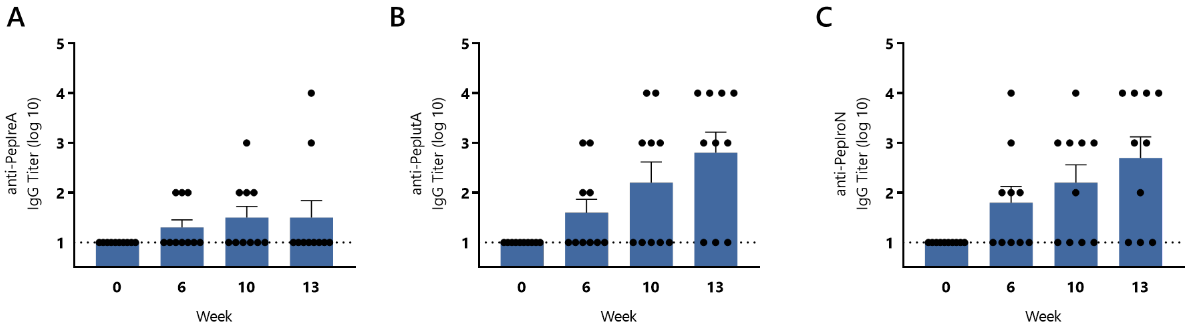

Figure S6: Individual epitope-specific antibody responses for mice from Fig. 5B. Antibody titers at week 13 titers were measured by ELISA against pIreA (A), pIutA (B), and pIroN (C).

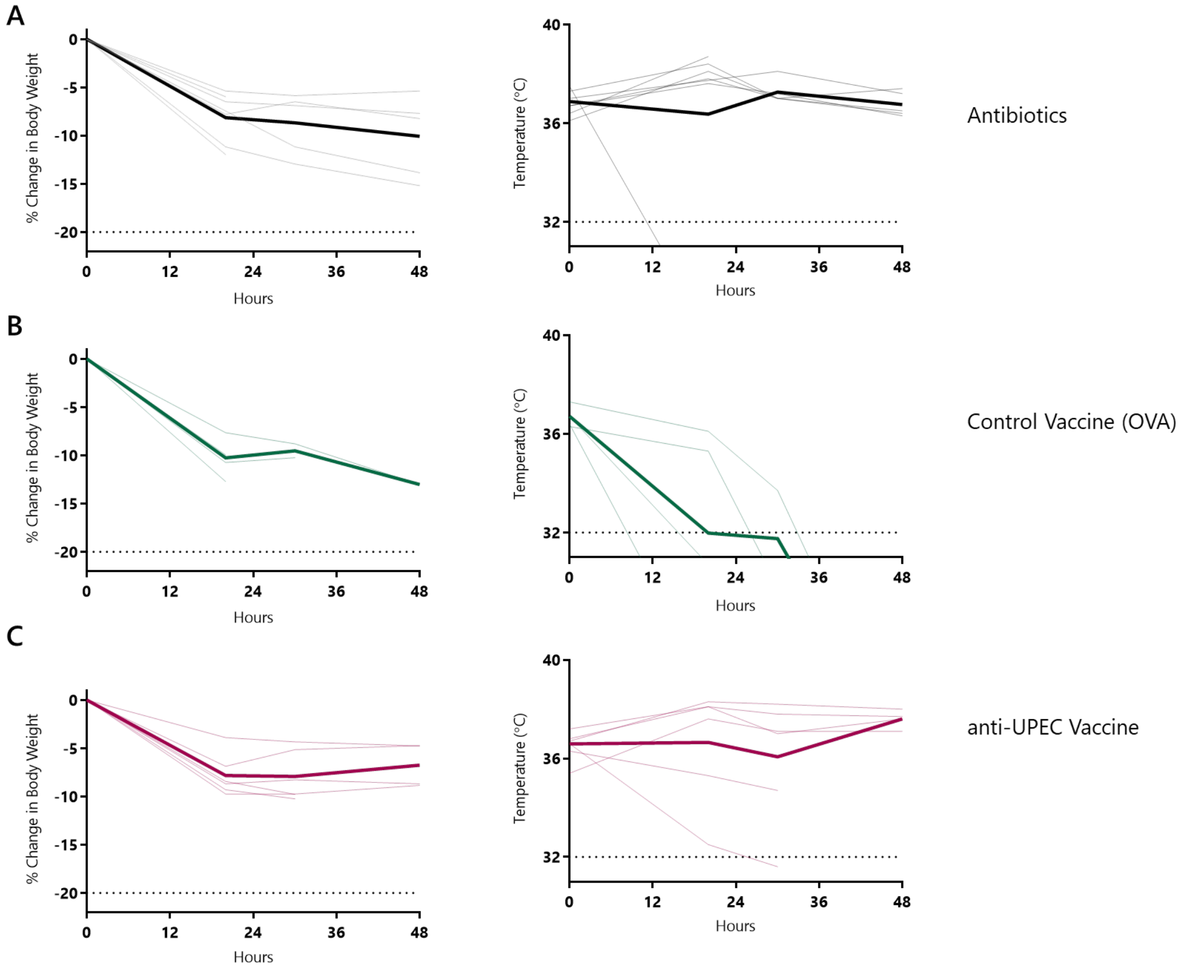

Figure S7: Body weight (left) and temperature (right) curves for individual mice in transurethral challenge in Figure 5A.

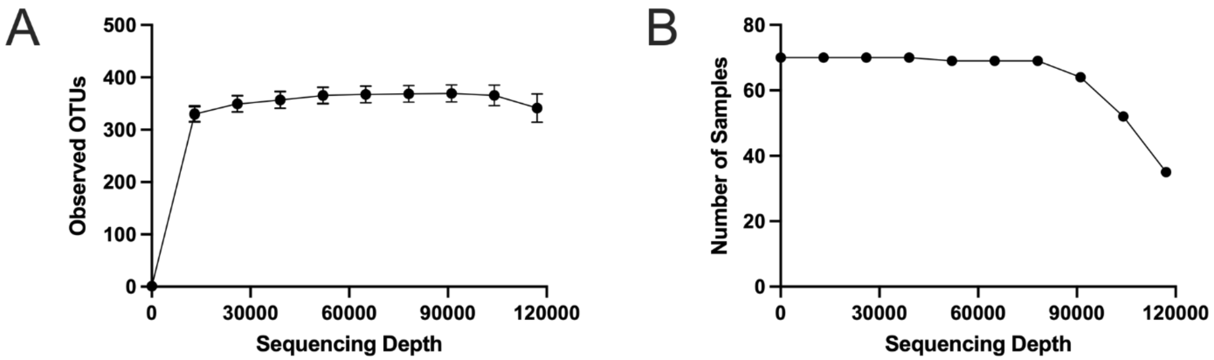

Figure S8: Richness sampling. Flattened curve in alpha rarefaction plot indicates the samples have been fully observed.

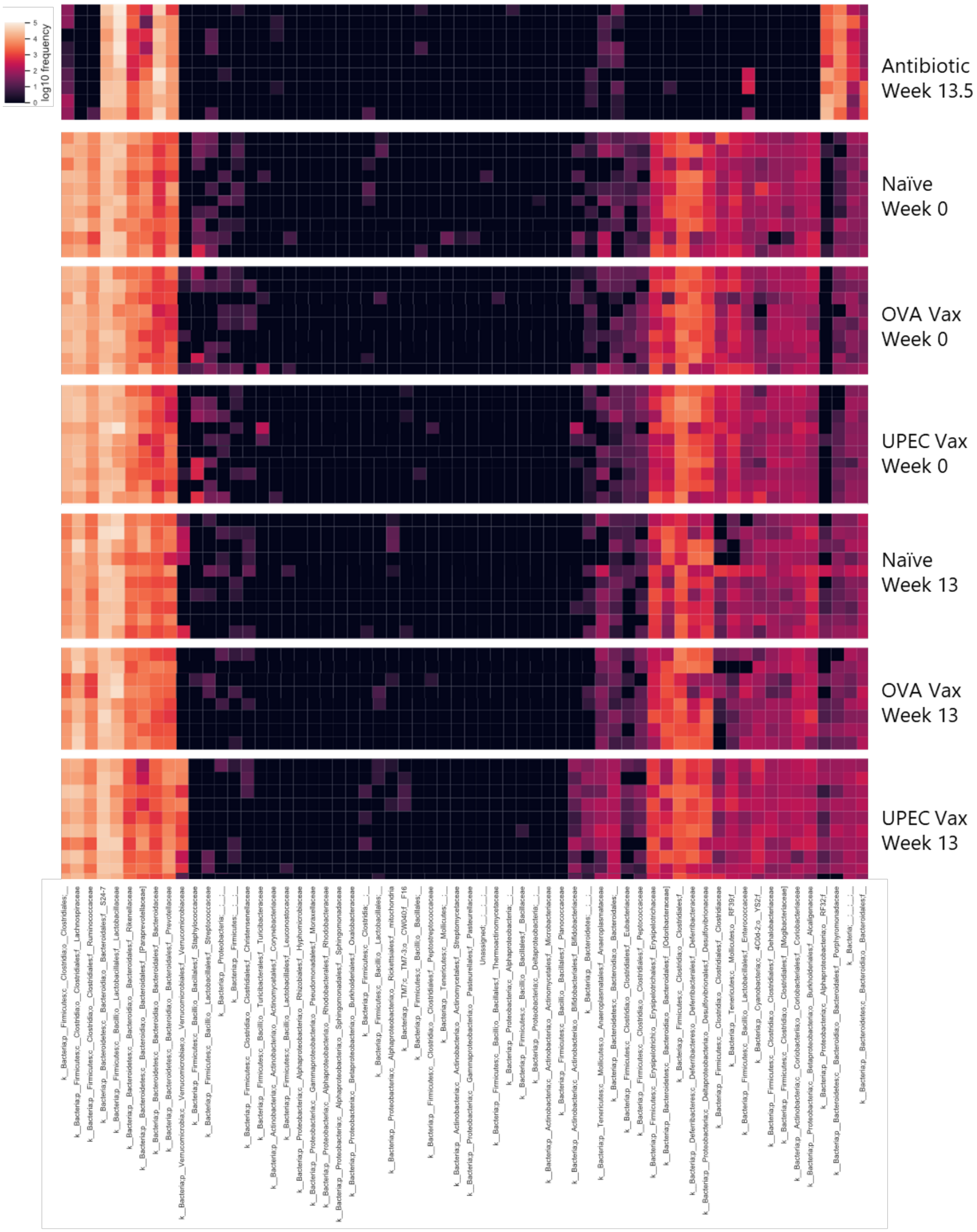

Figure S9: Complete family-level heatmap. Heatmap showing relative diversity of microbiome at the family taxonomic level. Each row represents an individual mouse.
